# Child psychiatric emergency visits during the COVID-19 pandemic

**DOI:** 10.1192/j.eurpsy.2022.1299

**Published:** 2022-09-01

**Authors:** J. Andreo Jover, D. Hernandez Calle, J. Curto-Ramos, L. Vicente Valor, D. García Martínez, G. Juárez, M. Alcamí, A. Ortiz, N. Iglesias, M.F. Bravo-Ortiz, G. Martinez-Ales, B. Rodríguez-Vega

**Affiliations:** 1 Universidad Autónoma de Madrid, Departament Of Psychiatry, Madrid, Spain; 2 Fundacion para la Investigación Biomédica del Hospital Universitario La Paz (IdiPAZ), Psychiatry, Madrid, Spain; 3 La Paz University Hospital, Madrid, Spain., Department Of Psychiatry, Clinical Psychology And Mental Health, Madrid, Spain; 4 La Paz University Hospital, Psychiatry, Clinical Psychology And Mental Health, Madrid, Spain; 5 Centro de Investigación Biomédica en Red de Salud Mental, Cibersam, Madrid, Spain; 6 Columbia University Mailman School of Public Health, Departament Of Epidemiology, Columbia, United States of America

**Keywords:** Urgency, Child, psychiatry, Covid-19

## Abstract

**Introduction:**

Paediatric and adult psychiatric emergency department (ED) visits decreased during the initial COVID-19 pandemic outbreak. Long-term consequences of the pandemic will include increases in mental healthcare needs especially among especially vulnerable groups such as children and adolescents.

**Objectives:**

This study examined changes in the number of overall and diagnosis-specific mental health ED visits among patients aged <18 years following onset of the COVID-19 pandemic in Madrid, Spain.

**Methods:**

We used electronic health records to extract the monthly numbers of total and diagnosis-specific mental health ED visits among patients aged <18 years, between October 2018 and April 2021, to La Paz University Hospital. We conducted interrupted time-series analyses and compared trends before and after the day of the first ED COVID-19 case (1^st^ March 2020).

**Results:**

In March 2020, there was a marked initial decrease of -12.8 (95%CI -21.9, -7.9) less monthly mental health ED visits. After April 2020, there was a subsequent increasing trend of 3.4 (95%CI 2.6, 4.2) additional monthly mental health ED visits.

**Conclusions:**

After onset of the COVID-19 pandemic, there was an increase in paediatric psychiatric ED visits, especially due to suicide-related reasons. These data reinforce the crucial role of the ED in the management of acute mental health problems among youth and highlight the need for renovated efforts to enhance access to care outside of and during acute crises during the pandemic and its aftermath.

**Disclosure:**

No significant relationships.

